# Characteristics of seasonal influenza A and B in Latin America: Influenza surveillance data from ten countries

**DOI:** 10.1371/journal.pone.0174592

**Published:** 2017-03-27

**Authors:** Saverio Caini, Wladimir J. Alonso, Angel Balmaseda, Alfredo Bruno, Patricia Bustos, Leticia Castillo, Celina de Lozano, Doménica de Mora, Rodrigo A. Fasce, Walquiria Aparecida Ferreira de Almeida, Gabriela F. Kusznierz, Jenny Lara, Maria Luisa Matute, Brechla Moreno, Claudio Maierovitch Pessanha Henriques, Juan Manuel Rudi, Clotilde El-Guerche Séblain, François Schellevis, John Paget, Global Influenza B Study group–Latin America

**Affiliations:** 1 Netherlands Institute for Health Services Research (NIVEL), Utrecht, The Netherlands; 2 Fogarty International Center, National Institutes of Health, Bethesda, Maryland, United States of America; 3 National Influenza Center, Ministry of Health, Managua, Nicaragua; 4 Instituto Nacional de Investigacion en Salud Publica (INSPI), Centro de Referencia Nacional de Influenza y Otros Virus Respiratorios, Guayaquil, Ecuador; 5 Seccion Virus Respiratorios, Instituto de Salud Publica de Chile, Santiago, Chile; 6 National Influenza Center, Ministry of Health, Guatemala City, Guatemala; 7 National Influenza Center, Ministry of Health, San Salvador, El Salvador; 8 Ministry of Health, Federal District, Brazil; 9 Instituto Nacional de Enfermedades Respiratorias “Dr. Emilio Coni”, ANLIS “C.Malbràn”, Santa Fe, Argentina; 10 National Influenza Center, Ministry of Health, San José, Costa Rica; 11 National Influenza Center, Ministry of Health, Tegucigalpa, Honduras; 12 National Influenza Center, IC Gorgas, Panama City, Panama; 13 Sanofi Pasteur, Lyon, France; 14 Department of General Practice and Elderly Care Medicine, EMGO Institute for Health Care Research VU University Medical Center, Amsterdam, The Netherlands; Columbia University, UNITED STATES

## Abstract

**Introduction:**

The increased availability of influenza surveillance data in recent years justifies an actual and more complete overview of influenza epidemiology in Latin America. We compared the influenza surveillance systems and assessed the epidemiology of influenza A and B, including the spatio-temporal patterns of influenza epidemics, in ten countries and sub-national regions in Latin America.

**Methods:**

We aggregated the data by year and country and characteristics of eighty-two years were analysed. We calculated the median proportion of laboratory-confirmed influenza cases caused by each virus strain, and compared the timing and amplitude of the primary and secondary peaks between countries.

**Results:**

37,087 influenza cases were reported during 2004–2012. Influenza A and B accounted for a median of 79% and, respectively, 21% of cases in a year. The percentage of influenza A cases that were subtyped was 82.5%; for influenza B, 15.6% of cases were characterized. Influenza A and B were dominant in seventy-five (91%) and seven (9%) years, respectively. In half (51%) of the influenza A years, influenza A(H3N2) was dominant, followed by influenza A(H1N1)pdm2009 (41%) and pre-pandemic A(H1N1) (8%). The primary peak of influenza activity was in June-September in temperate climate countries, with little or no secondary peak. Tropical climate countries had smaller primary peaks taking place in different months and frequently detectable secondary peaks.

**Conclusions:**

We found that good influenza surveillance data exists in Latin America, although improvements can still be made (e.g. a better characterization of influenza B specimens); that influenza B plays a considerable role in the seasonal influenza burden; and that there is substantial heterogeneity of spatio-temporal patterns of influenza epidemics. To improve the effectiveness of influenza control measures in Latin America, tropical climate countries may need to develop innovative prevention strategies specifically tailored to the spatio-temporal patterns of influenza in this region.

## Introduction

The study of the epidemiology of influenza and burden of disease has allowed countries to refine their strategies of control and prevention in the temperate climate regions of the world. Much less attention has been paid to influenza in the tropical climate countries, partly due to the belief that the burden of influenza is negligible when compared to other infectious diseases such as tuberculosis, malaria or AIDS. However, the burden of disease due to influenza has been found to be high in all countries where it has been measured, regardless of latitude [1–5]. In addition, the lack of reliable surveillance data has hindered a detailed study of the epidemiology of influenza A and B in the tropics, but this has changed in recent years, especially since the 2009 pandemic.

Several studies have recently compared the spatio-temporal patterns of influenza between tropical areas within large countries (e.g. Brazil [6–7], India [8] and China [9]) or within geographical regions in the tropics (e.g. south and south-eastern Asia [10] and Africa [11]). These studies have provided a better understanding of the climatic and demographic factors that trigger the onset and spread of influenza epidemics, and improved knowledge about the optimal timing for vaccination.

Latin America is a region with a population of 626 million people that contains much diversity in terms of topography and climate zones. Knowledge regarding the epidemiology of influenza has improved since the 2009 pandemic through an increased availability of good quality surveillance data; however, some aspects that may have profound implications for influenza prevention and control strategies still remain unclear and need to be examined in more depth.

Here, we compared the influenza surveillance systems and assessed the epidemiology of influenza A and B (including the spatio-temporal patterns of influenza epidemics) in ten countries in Latin America using the database of the Global Influenza B Study.

## Materials and methods

### Source of data

The Global Influenza B Study (GIBS) was launched in 2012 with the objective of producing scientific evidence needed to support the implementation of effective influenza prevention and control strategies worldwide. The methodology has been described in detail elsewhere [12]. Briefly, coordinators of national influenza surveillance systems of over fifty countries around the world (including countries situated in the Northern and Southern hemispheres and in the inter-tropical belt) were invited between 2013 and 2015 (depending on countries) to provide virological and epidemiological data on influenza that was collected within their national influenza surveillance system from 2000 until the date of contact. The GIBS has a focus on influenza B, but because data are collected for both influenza A and B, we can perform epidemiological analyses for both influenza virus types. The data received include the weekly number of reported laboratory-confirmed influenza cases (referred to as “cases” hereinafter) broken down by age group and influenza virus type and subtype/lineage and the weekly rates of influenza-like illness [ILI] or acute respiratory infection [ARI] (depending on availability). Thirty countries joined the GIBS as of December 2015. Participants were also required to describe their influenza surveillance system by answering a questionnaire specifically developed for GIBS. Consent was separately obtained in each country, when necessary, at the stage of data collection. No further permission is required to analyze the data. Contact information of data owners is available in S1 File.

**Fig 1:**
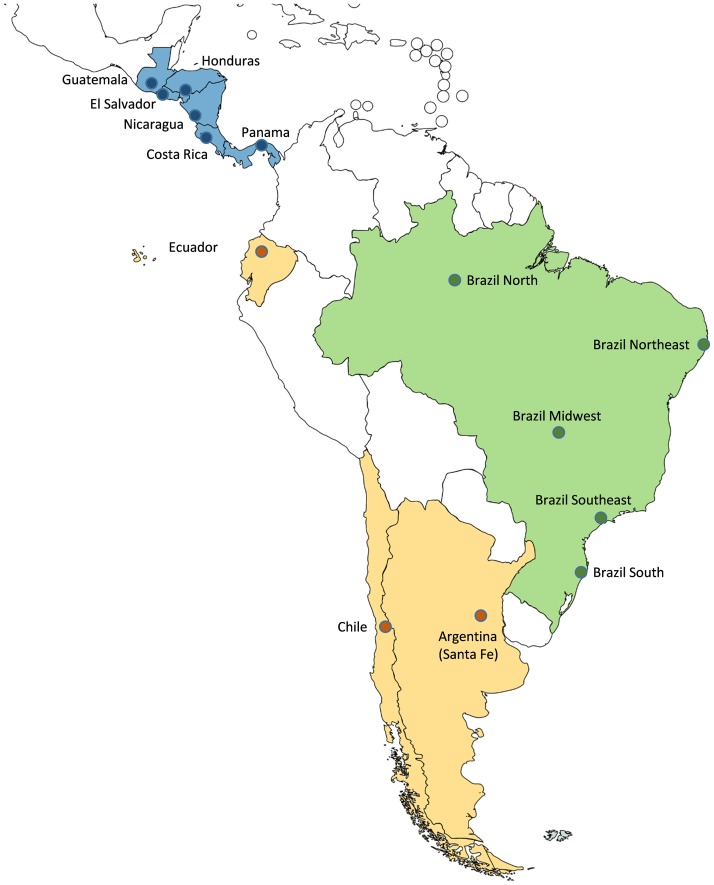
Countries and sub-national regions that were included in the analysis. **The Global Influenza B Study, Latin-American countries, 2003–2014**. Dark Blue: Central America. Light Blue: Brazil. Yellow: remaining sites in South America.

We included information on cases by virus type (A, B), subtype (H1N1, H1N1pdm09, H3N2, not subtyped) and lineage for influenza B (Victoria, Yamagata, non characterized) for the following sites (including countries and sub-national regions of Latin America): Province of Santa Fe in Argentina, Brazil Midwest, Brazil North, Brazil Northeast, Brazil South, Brazil Southeast, Chile, Costa Rica, Ecuador, El Salvador, Guatemala, Honduras, Nicaragua and Panama (Fig 1). Brazilian datasets included information on the virus type (A, B) but not on the subtype and lineage. Table 1 outlines the main features of the influenza surveillance systems that were included in the analysis.

**Table 1:** Main features of influenza surveillance systems of countries and sub-national regions included in the analysis. The Global Influenza B Study, Latin-American countries, 2003–2014.

Country	Latitude ^(1)^	Population (million)	Representativeness of data	% of outpatients ^(2)^	Laboratory methods for influenza diagnosis
**Guatemala**	14.6	15.4	National	72%	PCR, culture, immunofluorescence
**Honduras**	14.1	8.2	National	56%	PCR, culture, immunofluorescence
**El Salvador**	13.7	6.1	National	22%	PCR, culture, immunofluorescence
**Nicaragua**	12.1	6.1	National	43%	PCR, culture, immunofluorescence
**Costa Rica**	9.9	4.6	National	27%	PCR, culture, immunofluorescence
**Panama**	9.0	3.7	National	39%	PCR, culture, immunofluorescence
**Ecuador**	-0.2	15.4	National	0%	PCR
**Brazil North**	-3.1	15.0	National	unknown	PCR, immunofluorescence
**Brazil Northeast**	-8.1	53.1			
**Brazil Midwest**	-16.7	13.3			
**Brazil Southeast**	-23.6	80.4			
**Brazil South**	-27.6	27.4			
**Argentina (Santa Fe Province)**	-31.6	3.2	Regional	unknown	PCR, immunofluorescence
**Chile**	-33.5	16.6	National	5%	PCR, culture, immunofluorescence

^(1)^ Latitude of the country centroid (if available) or largest city.

^(2)^ Proportion of outpatients over all patients sampled.

### Measures and statistical analysis

We conducted an analysis using data aggregated by year and site. For each site, we initially included data from all calendar years with year-round influenza surveillance AND with at least 30 reported cases. We calculated the proportion of cases due to each influenza virus in each site and year. Monthly numbers of cases were standardized per year and divided by the highest number of cases in a month in the same site and year (hence, months with the highest number of cases for a given site and year were assigned the value 1); these value were then visualized by means of a heat map (in which the colour bar represents the intensity of monthly cases). We then calculated the median proportion of cases that were caused by each influenza virus (sub)type, and how frequently each of them caused the majority (50% or more) of all cases reported in a year.

We compared the timing and intensity of the primary and secondary influenza epidemic peaks between sites. For this analysis we excluded data from 2009, which was markedly atypical due to the introduction of a novel pandemic strain (A[H1N1]pdm2009). For each site, we first standardized each time series to proportions of the maximum annual value (so all years contribute equally). Next we generated a periodic annual function (PAF) of each time series by summing up the annual, semi-annual and quarterly harmonics as obtained by Fourier decomposition [13–14]. The timing and amplitude of the primary and secondary peaks of the PAF were extracted and compared between sites, as based on their latitudinal position. The timing of the primary and secondary peaks refers to the time of the year when the maximum influenza activity usually occurs. The amplitude of PAF is obtained by dividing the wave height (difference between the peak and trough values derived in the model) by the peak value. The peak amplitude is expressed as a percentage and must be interpreted as the intensity of the seasonality (when the signal is well defined); owing to how it is calculated, the amplitude can sometimes exceed 100%.

Analyses and figures were generated using the freely available analytical software Epipoi [14].

## Results

Data included in the present analysis originated from surveillance systems covering a population of about 270 million people, which corresponds to more than 40% of the total population of Latin America (Table 1). The proportion of outpatients among all sampled patients varied between 0% in Ecuador and 5% in Chile, to 56% in Honduras and 72% in Guatemala but respondents reported no significant changes in these percentages over time. All sites performed polymerase chain reaction (PCR) testing for influenza virus detections.

**Fig 2:**
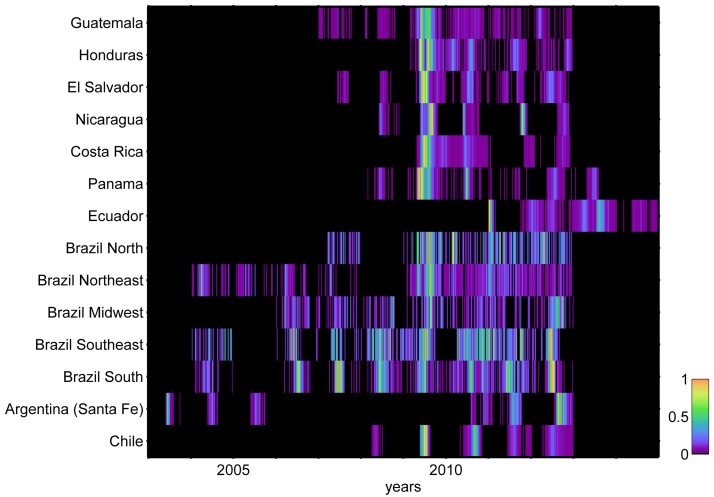
Heat map of monthly influenza virus incidence patterns, 2003–2014, sorted by latitude. The Global Influenza B Study, Latin-American countries, 2003–2014.

The total number of cases that were available for analysis was 37,087, of which 32,135 (86.6%) were influenza A and 4,952 (13.4%) were influenza B (Table 2). The percentage of influenza A cases that were subtyped was 82.5%; for influenza B, 15.6% of cases were characterized. The study period varied for each site, spanning from four years (Honduras, Costa Rica and Ecuador) to eight years of data (Brazil Northeast, Southeast and South) (median number of years of reporting = 6) (Fig 2). The median number of cases reported per year was 146 (range 31–4,072). The ILI rates were available only for Brazil (calculated as proportion of ILI over the total number of consultations) and Argentina (per 100,000 population) (Table 1).

**Table 2:** Laboratory-confirmed influenza cases by country and virus type, subtype and lineage. The Global Influenza B Study, Latin-American countries, 2003–2014.

Country	Years with data ^(a)^	No. cases	A(H1N1)	A(H1N1)pdm2009	A(H3N2)	A not subtyped	B Victoria	B Yamagata	B not characterized
Guatemala	2007–2012	**3,921**	308	1,708	377	1,053	0	0	475
Honduras	2009–2012	**1,701**	0	800	377	351	0	0	173
El Salvador	2007–2012	**2,271**	8	897	299	575	0	0	492
Nicaragua	2008–2012	**4,818**	141	2,888	638	442	0	0	709
Costa Rica	2009–2012	**6,083**	14	4,404	1,077	74	0	0	514
Panama	2008–2013	**2,191**	0	888	344	636	0	0	323
Ecuador	2011–2014	**1,872**	0	741	767	34	0	13	317
Brazil North	2007–2012	**332**	0	0	0	251	0	0	81
Brazil Northeast	2004–2012	**866**	0	0	0	620	0	0	246
Brazil Midwest	2006–2012	**403**	0	0	0	313	0	0	90
Brazil Southeast	2004–2012	**572**	0	0	0	418	0	0	154
Brazil South	2004–2012	**974**	0	0	0	694	0	0	280
Argentina (Santa Fe Province)	2003–2012	**607**	0	101	242	126	111	4	23
Chile	2008–2012	**10,476**	414	5,491	3,583	41	233	413	301
**Total**		**37,087**	**885 (2.4%)**	**17,918 (48.3%)**	**7,704 (20.8%)**	**5,628 (15.2%)**	**344 (0.9%)**	**430 (1.2%)**	**4,178 (11.3%)**

^(a)^ The following years were not included in the analysis because of fewer than 50 cases of influenza were reported: 2008 for Brazil North and Brazil Northeast, 2005 for Brazil Southeast and Brazil South, 2006–2009 for Argentina.

Cases were unevenly distributed across sites, years and strains. The top contributor was Chile (n = 10,476, corresponding 28.2% of all cases), with most of the cases from 2009 (38.9%). The pandemic strain contributed nearly half of the cases, followed by A(H3N2), A not-subtyped, B and pre-pandemic A(H1N1) (Table 2). The median percentage of influenza B was 20.5% (inter-quartile range 7.2%-35.3%), which rose to 23.2% (inter-quartile range 8.9%-37.2%) when ignoring the 2009 year. Influenza B was only rarely the dominant virus: this occurred in seven of 82 years (in 2008 in Brazil South, in 2010 in Brazil Midwest, South and Southeast, in 2011 in El Salvador, in 2012 in Nicaragua and Panama). There were 39 (out of 82) years in which A was the dominant virus and in which at least 20% of all influenza A cases were subtyped: A(H1N1) accounted for the majority of cases in three years (7.7% of all years), A(H1N1)pdm2009 in sixteen years (41.0%), and A(H3N2) in twenty years (51.3%). The number of cases by virus type, subtype and lineage in each year and site is available in the S1 Table.

**Fig 3:**
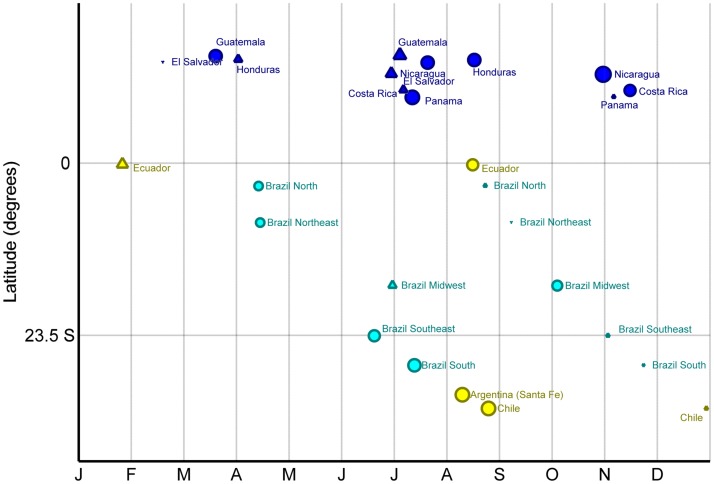
Timing and amplitude of primary (circles) and secondary (triangles) peak of influenza detection by site, against their latitudinal position. **The size corresponds to the amplitude of influenza seasonality**. Dark Blue: Central America. Light Blue: Brazil. Yellow: remaining sites in South America.

After excluding data from 2009, data of 20,525 cases were available for analysis on timing and intensity. The timing and amplitude of primary peaks of each site relative to their latitudes are shown in Table 3 and Fig 3. The timing of the primary peak of influenza activity was well aligned with the Southern Hemisphere (June-September) for sites located in the temperate climate region (latitude < -23.5: Chile, Argentina, Brazil South and Brazil Southeast). The timing of the primary peaks of influenza activity in the remaining sites (all within the tropical belt) had a more complex distribution throughout the year. The seasonality of influenza activity in Ecuador and in the Northern hemisphere countries of El Salvador, Panama and Honduras follows that of temperate climate regions in the Southern hemisphere (primary peak between beginning of July and end of August). The primary peak takes place in early October in Brazil Midwest, and in November in Nicaragua and Costa Rica. Finally, the North and Northeast regions of Brazil, Ecuador and Guatemala have their primary peak before May. The amplitude of the primary peak was above 90% in central-American countries (except Costa Rica, where it was 88.6%) and sites in the temperate climate region (except Brazil Southeast, where it was 80.5%), between 80% and 90% in Ecuador (88.6%), and below 80% in Brazil North, Northeast and Midwest (Table 3).

**Table 3:** Timing and amplitude of primary and secondary peaks of seasonal influenza epidemics. The Global Influenza B Study, Latin-American countries, 2003–2014.

Sites	Availability of data ^(a)^	Latitude (degrees)	Primary Peak		Secondary Peak	
			Timing (Month)	Amplitude	Timing (Month)	Amplitude
Guatemala	2007–2012	14.6	Mar	91.7%	Jul	82.9%
Honduras	2009–2012	14.1	Aug	96.8%	Apr	54.7%
El Salvador	2007–2012	13.7	Jul	99.4%	Feb	8.5%
Nicaragua	2008–2012	12.1	Nov	110.1%	Jun	72.8%
Costa Rica	2009–2012	9.9	Nov	88.6%	Jul	53.0%
Panama	2008–2013	9.0	Jul	107.8%	Nov	21.5%
Ecuador	2011–2014	-0.2	Aug	86.2%	Jan	75.1%
Brazil North	2007–2012	-3.1	Apr	63.5%	Aug	20.0%
Brazil Northeast	2004–2012	-8.1	Apr	61.3%	Sep	7.6%
Brazil Midwest	2006–2012	-16.7	Oct	73.8%	Jul	53.7%
Brazil Southeast	2004–2012	-23.6	Jun	80.5%	Nov	27.3%
Brazil South	2004–2012	-27.6	Jul	95.7%	Nov	17.2%
Argentina (Santa Fe)	2003–2012	-31.6	Aug	102.9%	-	0.0%
Chile	2008–2012	-33.5	Aug	104.5%	Dec	21.5%

^(a)^ Data from the pandemic year of 2009 was not included in the analyses

There were clear secondary peaks (amplitude > 70%) in Guatemala (taking place in early July), Nicaragua (late June) and Ecuador (late January) (Table 3 and Fig 3). There were milder yet clearly discernible secondary peaks (amplitude > 50%) in Honduras (March-April), Costa Rica (early July) and Brazil Midwest (end of June). The amplitude of secondary peaks was below 30% in all other sites.

## Discussion

We compared the main characteristics of influenza surveillance systems, assessed the relative contribution of the different virus (sub)types to influenza epidemics, and investigated the seasonal patterns of influenza circulation, in selected countries of Latin America (including sub-national data for Brazil) in recent years (2004–2014). The A(H3N2) virus strain was dominant during most years (except 2009), however influenza B played a considerable role in the seasonal influenza burden as it was responsible for around one fourth of all cases in a year on average. Finally, there was evidence of a substantial heterogeneity of both the amplitude and the timing of primary and secondary epidemic peaks across countries and sub-national regions in Latin America.

In the World Health Organization (WHO) Region of the Americas, influenza surveillance capacity has expanded following the 2009 pandemics, and the use of real-time PCR assays has become commonplace in the detection of influenza viruses in most countries. Despite considerable progress made in recent years, influenza surveillance is, however, not uniform throughout the region, and some gaps persist that need to be addressed by national and supranational health agencies. For instance, community- and hospital-based surveillance systems of respiratory pathogens (including influenza) should ideally be both in place everywhere as they both provide valuable yet complementary information [15–16]; it appears, however, that the former is still underdeveloped in several countries in the region. Unlike influenza A, a very limited percentage of influenza B cases were characterized. It should be noted though that since 2014 many NICs in Latin America have begun to characterize influenza B cases in a systematic way, after the US-CDC provided them with PCR assays for virus lineage differentiation, and this situation will likely improve in the coming years. Finally, denominator data were available for only two out of ten sites included in the study. Overcoming these limitations would allow an even better monitoring of influenza activity in Latin America and a more in-depth study of influenza patterns and disease burden, with potentially large public health impact.

Nearly half of all cases in our database were caused by the A(H1N1) pandemic strain. This was most likely a consequence of the increased sampling intensity during the pandemic: in effect, the majority of years were dominated by influenza A(H3N2), both before and after 2009. Influenza B was rarely the dominant virus, but it accounted for a quarter of all cases, which is in line with what has been reported in temperate countries of Northern and Southern hemispheres for the same period [12,17–19].

Our findings on the heterogeneity of influenza activity in Latin American countries do not necessarily affect the most recent recommendations on influenza vaccination (i.e. vaccinating in April using the Southern hemisphere formulation in all countries except Mexico and Cuba) [20]. However, it is critical to note that the proportion of cases preventable with a single dose of influenza vaccine administered in April-May will vary considerably between countries and across years within the same country. This observation appears even more evident when considering the poor temporal overlap of influenza A and B epidemics [21] and the rapid decline of vaccine-induced immunity during a single year [22–23]. Once the WHO goals for influenza vaccine coverage among vulnerable people are met, it will probably be necessary to reflect on what strategies could further reduce the burden of influenza in this part of Latin America, especially in tropical countries with two influenza activity peaks per year.

The identification of large areas encompassing countries or sub-national regions with a similar timing of influenza epidemics has implications for the organization of surveillance systems and the implementation of influenza vaccination campaigns. Overall, the partition into three large transmission zones that was made by WHO (Central America Caribbean from Mexico in the North to Panama in the South; Tropical South America from Colombia in the North to Peru, Bolivia and Brazil in the South; and Temperate South America including Chile, Argentina, Uruguay and Paraguay) [24] does not seem fully appropriate as it overlooks some of the differences we observed (for example, between regions in Brazil and between neighbouring countries in central America). Data from the literature for countries not included in our database [20, 25–27] provide further support for this view.

An important strength of our study is the availability of influenza surveillance data from countries and sub-national regions (for Brazil) spanning on a large range of latitudes and very diverse in terms of their geographical and climatic characteristics. Considering the uniform use of PCR, the potential bias of different laboratory testing biases are reduced. The use of statistical methods to account for high (or low) intensity years (e.g. the PAF) and the analysis of data by year also reduced the potential bias of larger years. Our study also has several limitations. The surveillance systems differ across countries, in particular for what concerns the percentage of in- and out-patients; however, this should not affect the results of our spatio-temporal analysis, because the timing of virus activity is likely to be similar for mild and severe cases. Influenza surveillance data was available for a limited number of years for some sites. The epidemiological parameters (timing and amplitude of primary and secondary peak) that we estimated may change by extending the study period by a few years, although we believe that this would not lead to major changes in our conclusions. Information on lineage was available for only a few influenza B cases, which prevented us from assessing the level of co-circulation of both lineages and the frequency of influenza B lineage-level vaccine mismatch. Finally, we had no data available for some large and densely populated countries like Colombia, Venezuela and Peru.

In conclusion, the substantial heterogeneity of spatio-temporal patterns of influenza epidemics between countries of Latin America, especially those located in tropical regions, implies that the effectiveness of the annual seasonal influenza vaccine may be moderate or low in some countries and some years. In tropical countries or regions where there is little seasonality and/or bimodal epidemic curves, effectiveness may be consistently low because protection may not have time to develop, or have already fallen, when influenza activity peaks. These latter findings call for a reflection on how to move beyond the limits of applying existing influenza prevention strategies that have proved effective in temperate climates to tropical areas of Latin America.

## Supporting information

S1 Table Laboratory-confirmed influenza cases reported to the local influenza surveillance system in each year and country or sub-national region, by virus type, subtype and lineage. The Global Influenza B Study, Latin-American countries, 2004–2014.
(DOC)

S1 File Contact information of data owners. (DOC)

## Abbreviations

ARI: Acute Respiratory Infection
GIBS: Global Influenza B Study
ILI: Influenza-like Illness
PAF: Periodic Annual Function
WHO: World Health Organization
