# Supplementary material for: Characteristics of seasonal influenza A and B in Latin America: Influenza surveillance data from ten countries
Source: PLoS One. 2017 Mar 27;12(3):e0174592. doi: 10.1371/journal.pone.0174592 (PMC5367818; doi:10.1371/journal.pone.0174592)
Supplement: S1 Table — The Global Influenza B Study, Latin-American countries, 2004–2014. (DOC) [file pone.0174592.s001.doc]

| **Country** | **Year** | **No. cases** | **A(H1N1)** | **A(H1N1)pdm2009** | **A(H3N2)** | **A not subtyped** | **B Victoria** | **B Yamagata** | **B not characterized** |
| --- | --- | --- | --- | --- | --- | --- | --- | --- | --- |
| Guatemala | 2007 | 219 | 0 | 0 | 13 | 205 | 0 | 0 | 1 |
| Guatemala | 2008 | 185 | 41 | 0 | 25 | 56 | 0 | 0 | 63 |
| Guatemala | 2009 | 2268 | 267 | 1,308 | 174 | 411 | 0 | 0 | 108 |
| Guatemala | 2010 | 584 | 0 | 223 | 90 | 63 | 0 | 0 | 208 |
| Guatemala | 2011 | 285 | 0 | 3 | 68 | 185 | 0 | 0 | 29 |
| Guatemala | 2012 | 380 | 0 | 174 | 7 | 133 | 0 | 0 | 66 |
| Honduras | 2009 | 904 | 0 | 549 | 4 | 334 | 0 | 0 | 17 |
| Honduras | 2010 | 405 | 0 | 155 | 172 | 0 | 0 | 0 | 78 |
| Honduras | 2011 | 222 | 0 | 23 | 122 | 15 | 0 | 0 | 62 |
| Honduras | 2012 | 170 | 0 | 73 | 79 | 2 | 0 | 0 | 16 |
| El Salvador | 2007 | 88 | 0 | 0 | 0 | 87 | 0 | 0 | 1 |
| El Salvador | 2008 | 88 | 0 | 0 | 0 | 55 | 0 | 0 | 33 |
| El Salvador | 2009 | 1098 | 4 | 659 | 7 | 410 | 0 | 0 | 18 |
| El Salvador | 2010 | 352 | 3 | 20 | 189 | 19 | 0 | 0 | 121 |
| El Salvador | 2011 | 202 | 1 | 6 | 93 | 1 | 0 | 0 | 101 |
| El Salvador | 2012 | 443 | 0 | 212 | 10 | 3 | 0 | 0 | 218 |
| Nicaragua | 2008 | 390 | 85 | 0 | 0 | 153 | 0 | 0 | 152 |
| Nicaragua | 2009 | 2404 | 56 | 2,153 | 23 | 172 | 0 | 0 | 0 |
| Nicaragua | 2010 | 646 | 0 | 11 | 353 | 63 | 0 | 0 | 219 |
| Nicaragua | 2011 | 928 | 0 | 719 | 147 | 53 | 0 | 0 | 9 |
| Nicaragua | 2012 | 450 | 0 | 5 | 115 | 1 | 0 | 0 | 329 |
| Costa Rica | 2009 | 3898 | 14 | 3,545 | 202 | 57 | 0 | 0 | 80 |
| Costa Rica | 2010 | 1441 | 0 | 762 | 491 | 12 | 0 | 0 | 176 |
| Costa Rica | 2011 | 194 | 0 | 49 | 118 | 0 | 0 | 0 | 27 |
| Costa Rica | 2012 | 550 | 0 | 48 | 266 | 5 | 0 | 0 | 231 |
| Panama | 2008 | 119 | 0 | 13 | 0 | 79 | 0 | 0 | 27 |
| Panama | 2009 | 1270 | 0 | 744 | 3 | 442 | 0 | 0 | 81 |
| Panama | 2010 | 328 | 0 | 8 | 173 | 91 | 0 | 0 | 56 |
| Panama | 2011 | 59 | 0 | 43 | 2 | 9 | 0 | 0 | 5 |
| Panama | 2012 | 243 | 0 | 64 | 18 | 7 | 0 | 0 | 154 |
| Panama | 2013 | 172 | 0 | 16 | 148 | 8 | 0 | 0 | 0 |
| Ecuador | 2011 | 466 | 0 | 140 | 315 | 11 | 0 | 0 | 0 |
| Ecuador | 2012 | 410 | 0 | 47 | 168 | 10 | 0 | 0 | 185 |
| Ecuador | 2013 | 838 | 0 | 533 | 230 | 2 | 0 | 0 | 73 |
| Ecuador | 2014 | 158 | 0 | 21 | 54 | 11 | 0 | 13 | 59 |
| Brazil North | 2007 | 34 | 0 | 0 | 0 | 28 | 0 | 0 | 6 |
| Brazil North | 2009 | 95 | 0 | 0 | 0 | 75 | 0 | 0 | 20 |
| Brazil North | 2010 | 51 | 0 | 0 | 0 | 40 | 0 | 0 | 11 |
| Brazil North | 2011 | 77 | 0 | 0 | 0 | 48 | 0 | 0 | 29 |
| Brazil North | 2012 | 75 | 0 | 0 | 0 | 60 | 0 | 0 | 15 |
| Brazil Northeast | 2004 | 85 | 0 | 0 | 0 | 83 | 0 | 0 | 2 |
| Brazil Northeast | 2005 | 46 | 0 | 0 | 0 | 42 | 0 | 0 | 4 |
| Brazil Northeast | 2006 | 53 | 0 | 0 | 0 | 45 | 0 | 0 | 8 |
| Brazil Northeast | 2007 | 31 | 0 | 0 | 0 | 25 | 0 | 0 | 6 |
| Brazil Northeast | 2009 | 278 | 0 | 0 | 0 | 201 | 0 | 0 | 77 |
| Brazil Northeast | 2010 | 115 | 0 | 0 | 0 | 70 | 0 | 0 | 45 |
| Brazil Northeast | 2011 | 175 | 0 | 0 | 0 | 101 | 0 | 0 | 74 |
| Brazil Northeast | 2012 | 83 | 0 | 0 | 0 | 53 | 0 | 0 | 30 |
| Brazil Midwest | 2006 | 55 | 0 | 0 | 0 | 43 | 0 | 0 | 12 |
| Brazil Midwest | 2007 | 43 | 0 | 0 | 0 | 32 | 0 | 0 | 11 |
| Brazil Midwest | 2008 | 45 | 0 | 0 | 0 | 28 | 0 | 0 | 17 |
| Brazil Midwest | 2009 | 74 | 0 | 0 | 0 | 65 | 0 | 0 | 9 |
| Brazil Midwest | 2010 | 46 | 0 | 0 | 0 | 22 | 0 | 0 | 24 |
| Brazil Midwest | 2011 | 43 | 0 | 0 | 0 | 33 | 0 | 0 | 10 |
| Brazil Midwest | 2012 | 97 | 0 | 0 | 0 | 90 | 0 | 0 | 7 |
| Brazil Southeast | 2004 | 44 | 0 | 0 | 0 | 29 | 0 | 0 | 15 |
| Brazil Southeast | 2006 | 41 | 0 | 0 | 0 | 31 | 0 | 0 | 10 |
| Brazil Southeast | 2007 | 36 | 0 | 0 | 0 | 32 | 0 | 0 | 4 |
| Brazil Southeast | 2008 | 90 | 0 | 0 | 0 | 62 | 0 | 0 | 28 |
| Brazil Southeast | 2009 | 88 | 0 | 0 | 0 | 84 | 0 | 0 | 4 |
| Brazil Southeast | 2010 | 106 | 0 | 0 | 0 | 53 | 0 | 0 | 53 |
| Brazil Southeast | 2011 | 100 | 0 | 0 | 0 | 64 | 0 | 0 | 36 |
| Brazil Southeast | 2012 | 67 | 0 | 0 | 0 | 63 | 0 | 0 | 4 |
| Brazil South | 2004 | 51 | 0 | 0 | 0 | 47 | 0 | 0 | 4 |
| Brazil South | 2006 | 94 | 0 | 0 | 0 | 71 | 0 | 0 | 23 |
| Brazil South | 2007 | 129 | 0 | 0 | 0 | 123 | 0 | 0 | 6 |
| Brazil South | 2008 | 127 | 0 | 0 | 0 | 59 | 0 | 0 | 68 |
| Brazil South | 2009 | 113 | 0 | 0 | 0 | 92 | 0 | 0 | 21 |
| Brazil South | 2010 | 147 | 0 | 0 | 0 | 67 | 0 | 0 | 80 |
| Brazil South | 2011 | 175 | 0 | 0 | 0 | 129 | 0 | 0 | 46 |
| Brazil South | 2012 | 138 | 0 | 0 | 0 | 106 | 0 | 0 | 32 |
| Argentina (Santa Fe Province) | 2003 | 67 | 0 | 0 | 46 | 21 | 0 | 0 | 0 |
| Argentina (Santa Fe Province) | 2004 | 45 | 0 | 0 | 26 | 19 | 0 | 0 | 0 |
| Argentina (Santa Fe Province) | 2005 | 53 | 0 | 0 | 14 | 24 | 4 | 1 | 10 |
| Argentina (Santa Fe Province) | 2010 | 50 | 0 | 0 | 15 | 20 | 15 | 0 | 0 |
| Argentina (Santa Fe Province) | 2011 | 144 | 0 | 6 | 119 | 19 | 0 | 0 | 0 |
| Argentina (Santa Fe Province) | 2012 | 248 | 0 | 95 | 22 | 23 | 92 | 3 | 13 |
| Chile | 2008 | 478 | 403 | 0 | 0 | 40 | 7 | 2 | 26 |
| Chile | 2009 | 4072 | 11 | 4,037 | 21 | 1 | 0 | 0 | 2 |
| Chile | 2010 | 2934 | 0 | 618 | 2,020 | 0 | 24 | 5 | 267 |
| Chile | 2011 | 1095 | 0 | 825 | 259 | 0 | 7 | 0 | 4 |
| Chile | 2012 | 1897 | 0 | 11 | 1,283 | 0 | 195 | 406 | 2 |
