# Supplementary material for: Characteristics of seasonal influenza A and B in Latin America: Influenza surveillance data from ten countries
Source: PLoS One. 2017 Mar 27;12(3):e0174592. doi: 10.1371/journal.pone.0174592 (PMC5367818; doi:10.1371/journal.pone.0174592)
Supplement: S1 File — (DOC) [file pone.0174592.s002.doc]

These datasets are owned by the participating countries, and thus cannot be shared publicly by the study group at NIVEL. Researchers interested in obtaining the country-specific datasets may contact the individuals listed in the Supporting Information for further details regarding data access.

**Argentina (Santa Fe Province)**: Gabriela Kusznierz ([labconi@yahoo.com.ar](mailto:labconi@yahoo.com.ar))

**Brazil**: Jose Ricardo Pio Marins ([ricardo.marins@saude.gov.br](mailto:ricardo.marins@saude.gov.br))

**Chile**: Rodrigo Fasce ([rfasce@ispch.cl](mailto:rfasce@ispch.cl))

**Costa** **Rica**: Alexey W. Clara ([wclara@cdc.gov](mailto:wclara@cdc.gov))

**Ecuador**: Alfredo Bruno ([alfredobruno@yahoo.es](mailto:alfredobruno@yahoo.es))

**El** **Salvador**: Alexey W. Clara ([wclara@cdc.gov](mailto:wclara@cdc.gov))

**Guatemala**: Alexey W. Clara ([wclara@cdc.gov](mailto:wclara@cdc.gov))

**Honduras**: Alexey W. Clara ([wclara@cdc.gov](mailto:wclara@cdc.gov))

**Nicaragua**: Alexey W. Clara ([wclara@cdc.gov](mailto:wclara@cdc.gov))

**Panama**: Alexey W. Clara ([wclara@cdc.gov](mailto:wclara@cdc.gov))
